# Characterisation of assembly and ubiquitylation by the RBCC motif of Trim5α

**DOI:** 10.1038/srep26837

**Published:** 2016-05-27

**Authors:** Jeremy R. Keown, Joy X. Yang, Jordan Douglas, David C. Goldstone

**Affiliations:** 1 School of Biological Sciences, University of Auckland, Auckland, New Zealand; 2 Associate Investigator Maurice Wilkins Centre for Molecular Biodiscovery, New Zealand

## Abstract

The post-entry restriction factor Trim5α blocks infection of retroviral pathogens shortly after the virus gains entry to the cell, preventing reverse transcription and integration into the host genome. Central to the mechanism of restriction is recognition of the lattice of capsid protein that forms the inner-shell of the retrovirus. To recognise this lattice, Trim5α has been shown to assemble into a large hexagonal array, complementary to the capsid lattice. Structures of the Trim5α coiled-coil region reveal an elongated anti-parallel dimer consistent with the edges of this array placing the Bbox domain at each end of the coiled-coil to facilitate assembly. To investigate the nature of this assembly we have designed and characterised a monomeric version of the TRIM RBCC motif with a truncated coiled-coil. Biophysical characterisation by SEC-MALLS, AUC, and SAXS demonstrate that this construct forms compact folded domain that assembles into a trimer that would support the formation of a hexagonal lattice. Furthermore, the RING domain and elements of the coiled-coil region are shown to contribute to assembly. Ubiquitylation assays demonstrate that this assembly increases ubiquitylation activity providing a link from recognition of the capsid lattice and assembly to the activation of innate immune signalling and restriction.

Mammalian cells possess intrinsic defences against retroviral pathogens. These responses to infection are facilitated by restriction factors that act during multiple stages of the retroviral life cycle to recognise, prevent, and contain infection. Such restriction factors include SamHD1^1^, the Apobec3^2^ and Mx proteins^3^, and members of the TRIM protein family.

Trim5α is one such factor that acts during the early post-entry stages of the retroviral lifecycle, blocking reverse transcription and integration into the host genome^4^. Like all members of the TRIM family, Trim5α has a conserved N-terminal domain architecture; comprising a RING domain, a Bbox domain, and coiled-coil motif^5^. Additionally, the C-terminal domain of Trim5α is a PRY/SPRY domain.

While the mechanism of restriction is poorly understood, there are at least two blocks to infection. The first block, prior to reverse transcription, is dependent upon the ubiquitin proteasome system and results in premature disassembly of the capsid core and degradation of viral proteins and RNA^6^. Inhibition of the proteasome rescues reverse transcription but is unable to rescue infection^7^. A similar result is obtained if ubiquitylation is blocked by removing or disrupting the Trim5α RING domain^8^.

In addition to direct effects on the incoming virus Trim5α also activates innate immune signalling via the production of ubiquitin chains. This results in the activation of TAK1 and downstream inflammatory pathways. In this capacity Trim5α acts as a pattern recognition receptor for the assembled retroviral capsid^9^. More recently the autophagy machinery has been implicated in Trim5α mediated restriction in a report suggesting Trim5α acts as a selective autophagy receptor^10^.

Central to the mechanism of restriction is recognition of the incoming retroviral capsid. Trim5α restricts retroviral infection in a species-specific manner with the Trim5α orthologue from an individual species able to restrict a subset of retroviruses^11^. The C-terminal PRY/SPRY domain of Trim5α is responsible for the recognition of a particular retrovirus through residues located in four variable loops^12^. Recognition occurs by direct interaction with the retroviral capsid protein; however, this interaction is weak and a single capsid protein is insufficient to trigger restriction^13^. Instead, the intact lattice of capsid protein that forms the inner shell of the retrovirus is required^14^,^15^.

To recognise the capsid lattice Trim5α has been shown to assemble into a large hexagonal array complementary to the retroviral capsid lattice^16^. To investigate the nature of this assembly and the interactions that govern its formation we have undertaken a structural and biophysical approach, examining the self-association of Trim5α to characterise the interfaces that are required for the assembly.

We have previously determined the structure of the Bbox and coiled-coil regions from Rhesus macaque Trim5α^17^. This structure showed the coiled-coil region to be a dimeric, elongated anti-parallel assembly with a length consistent with the edges of the Trim5α lattice seen in published electron micrographs^16^. The antiparallel assembly places the Bbox domain of each monomer at opposite ends of the coiled-coil suggesting they are responsible for mediating formation of three fold vertices at the corners of the lattice. This model is consistent with experiments where mutations in the Bbox domain block higher-order assembly of Trim5α^18^.

In this paper we use SEC-MALLS, SAXS, AUC, and ubiquitylation assays to show that the elements of the antiparallel coiled-coil, and the L1 region between the RING and Bbox domains, contribute to Bbox-mediated assembly at the three-fold interface. Furthermore, we show that this assembly increases ubiquitylation activity via the Trim5α RING domain.

## Results

### The Bbox domain shows weak self-association characteristics

**Figure 1:**
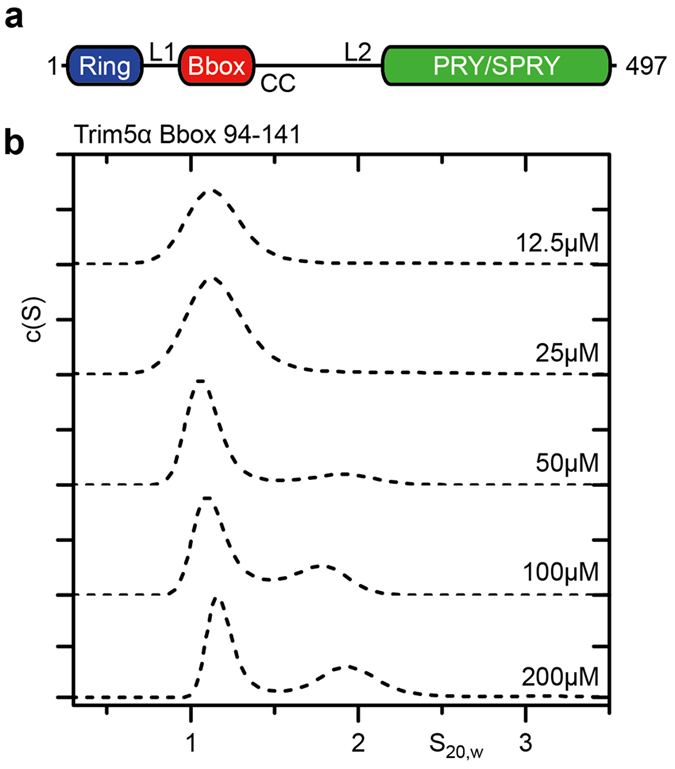
The Trim5α Bbox domain shows weak self-association. (**a**) Diagram of the Trim5α domain arrangement. (**b**) Sedimentation velocity c(S) analysis of RhT5α 94–141 over a concentration range of 12.5 μM and 200 μM. Normalised c(S) profiles for each concentration are shown. The emergence of a second peak is evident above 50 μM.

The antiparallel nature of the Trim5α coiled-coil places the Bbox domains at opposing ends of the coiled coil. This causes difficulties in experimentally dissecting the assembly of Trim5α as binding interfaces at each end of the coiled-coil result in polymers that preclude the characterization of a single binding interface. Therefore, to examine the assembly of Trim5α we first undertook an analysis of the Bbox domain alone (residues 94–141). The structure of the Human Trim5α Bbox domain has previously been determined by NMR and shown to form a weak dimer^18^. At low protein concentrations (12.5–25 μM) sedimentation velocity AUC experiments of RhT5 94–141 show a single broad peak at ~1.2 S in the c(s) analysis (Fig. 1, Table S1). However, as the protein concentration increases a second fast peak at ~1.7 S appears, revealing a concentration dependent oligomerisation. At a protein concentration of 200 μM, the highest concentration measured, 41.7% of the total signal was present in the second peak. These proportions suggest a half point of association in the high micro-molar range. Analysis of the 200 μM data set suggests molecular weights of 8.7 and 18.3 kDa for the first and second peaks respectively. Exact molecular weight and oligomer determination was not possible due to the mixture of species in the sample and low abundance of the larger species. However this suggests a monomer-dimer transition similar to that described previously^18^. Unfortunately, attempts to characterise the association using sedimentation equilibrium AUC were unsuccessful due to insufficient protein stability.

### Design and expression of truncated coiled-coil constructs

**Figure 2:**
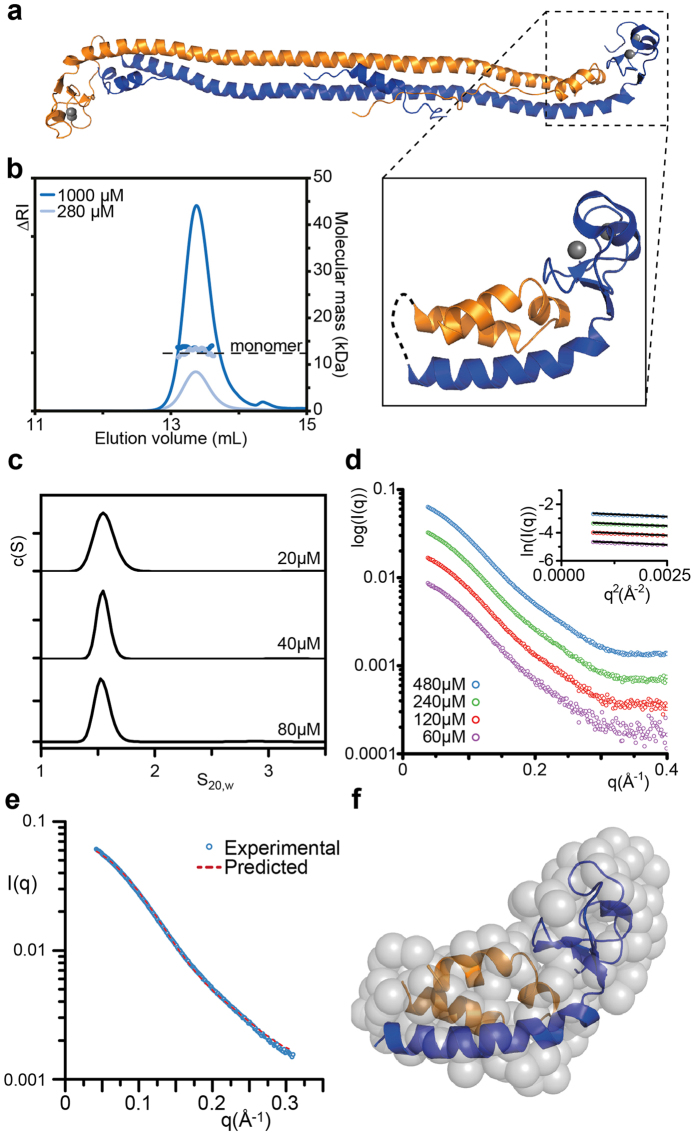
The Bbox and elements of the coiled-coil fold as a single domain. Design and characterization of RhT5α 88–261dCC EK/RD. (**a**) Structure of the Bbox-coiled-coil region from Trim5α (PDB id:4TN3^17^) Predicted structure of RhT5 88–261dCC with designed linker shown as dashed line is shown inset. Residues from monomer A are coloured blue and monomer B are coloured orange. Zinc atoms are shown as grey spheres. (**b)** SEC-MALLS analysis. Chromatogram from the differential refractive index detector are shown at two protein concentrations. M_w_ measured across the peak corresponding to a monomeric species is shown as open circles. (**c**) Sedimentation velocity AUC c(S) analysis at three protein concentrations. (**d**) SAXS curves between 60–480 μM with inset guinier analysis. (**e**) Comparison of SAXS scattering at 480 μM (6 mg/ml) to predicted model shown in (**a**). (**f**) *ab initio* bead model from SAXS generated in GASBOR compared to the predicted model shown in (**a**).

The AUC analysis of our Bbox construct as well as published NMR and AUC experiments^18^ suggest the isolated Bbox domain assembles weakly as a dimer in solution. This observation is at odds with the observed hexagonal assembly of Trim5–21 R chimeras where a trimeric interaction would be required for assembly^16^. Examination of our Trim5α Bbox-coiled-coil structure reveals a cluster of hydrophobic residues on one face of the central β-sheet of the Bbox domain^17^. This hydrophobic cluster is protected by the hairpin turn of the coiled-coil, shielding it from solvent and forming an extended hydrophobic core with elements of the hairpin. We hypothesised that the exposure of this patch on previously studied constructs, comprising residues solely from the Bbox domain, contributed to low protein stability and very weak associative properties. To stabilise the Bbox domain and maintain interactions present in full length Trim5α we designed constructs that included these elements from the coiled-coil region. Our construct comprises residues Pro88-Lys154 (Bbox-CC) and Tyr229-Asn261 (CC-L2) from the opposing monomer. A four residue linker (GGDP) links residues Lys154 from monomer A and Tyr229 from monomer B within the truncated coiled coil domain (Fig. 2a). The short linker is designed based on truncation mutants of MxA^19^ to terminate the helix from monomer A and make a short loop before initiating the start of the second helix in the opposite direction. The utility of this construct design is to create a monomeric version of the Trim5α Bbox-coiled-coil interface that will allow biophysical characterisation of a single Bbox interface and therefore Trim5α assembly without the formation of higher-order polymers.

To validate our approach and examine whether the construct (RhT5 88–261dCC) folded as expected, we first tested a construct that included the E120K/R121D mutation (RhT5 88–261dCC EK/RD) that has previously been shown to block higher order assembly of the Bbox domain^17^,^18^. This construct was expressed and purified from *E. coli* and subjected to size-exclusion chromatography coupled to multi-angle laser light scattering (SEC-MALLS), sedimentation velocity-AUC (SV-AUC), and small angle X-ray scattering (SAXS) analysis. SEC-MALLS analysis showed the protein to elute as a single symmetric peak with a retention volume of 13.5 mL and a M_*w*_ of 12,500 Da in agreement with the monomer Mr of 12,354 Da. Additionally, there was no concentration dependence in either elution position or M_w_, characteristic of a single monomeric species in solution (Fig. 2b). SV-AUC of RhT5 88–261dCC EK/RD showed the protein to sediment as a single species with an S_20,w_ of 1.55 S across a concentration range of 20–80 μM (Fig. 2c, Table S2).

SAXS data of RhT5 88–261dCC EK/RD were collected across a concentration range of 0.75 to 6.0 mg/mL (60–480 μM) and also showed no concentration dependence in either the intensity at zero angle (I(0)) or the radius of gyration (Rg). Guinier analysis gave an Rg of 17.3 Å, while the pair-distribution function is consistent with a compact folded protein with a D_max_ of 60 Å (Fig. 2d, Fig. S3 and Table S4). These values are in agreement with theoretical values calculated using the program HYDROPRO^20^ and our predicted model (D_max_ 59 Å, Rg 16.6 Å)^20^. Furthermore, comparison of the scattering with our predicted model of RhT5 88–261dCC using CRYSOL^21^ gave excellent agreement across a Q-range of 0.0005–0.3 Å^−1^ with a Chi-square value of 0.776 (Fig. 2e). *Ab initio* bead models calculated from the scattering curves using GASBOR are in good agreement with the predicted structure (Fig. 2f).

Based upon the results of these experiments we conclude that the construct behaved as expected resulting in a single monomeric species consistent with our model and that the linker maintained the structural elements of the coiled-coil in the appropriate arrangement.

### The Trim5α coiled-coil promotes formation of a trimer

**Figure 3:**
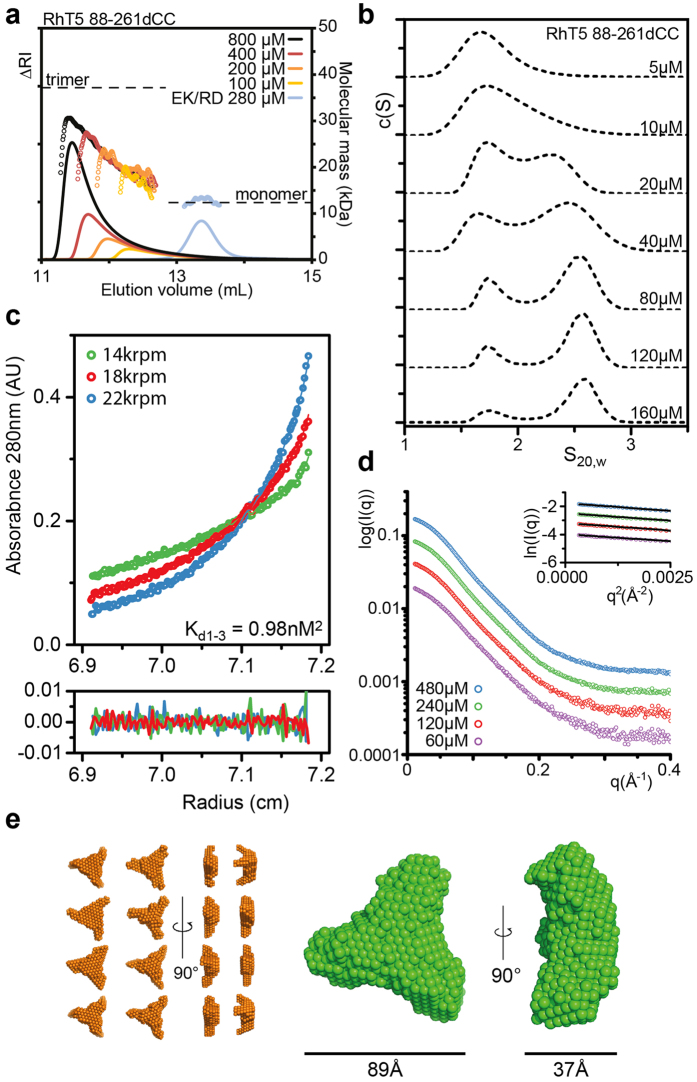
Trim5α coiled-coil contributes to assembly (**a**) SEC-MALLS analysis of RhT5 88–261dCC shows an increase in elution position and M_w_ with increasing concentration. (**b**) SV-AUC c(S) analysis of RhT5 88–261dCC over a concentration range of 5–160 μM. Normalised c(S) profiles are shown. (**c**) (upper) Multispeed SE-AUC of RhT5 88–261dCC. Data were collected at three starting concentrations and fit taking into account time and radially independent noise. A single starting concentration (20 μM) is shown. (lower) Residuals of the fit to a monomer-trimer equilibrium with dissociation constant of 0.98 nM^2^. (**d**) SAXS curves between 60–480 μM with guinier curves inset. (**e**) *ab initio* models of the RhT5 88–261dCC trimer were produced using DAMMIF^36^. Selected raw models are shown in orange, consensus averaged model generated in DAMAVER is shown in green.

To investigate the assembly of Trim5α we next examined the RhT5 88–261dCC construct lacking the assembly blocking mutations. Wild-type RhT5 88–261dCC also eluted from the SEC-MALLS as a single peak in the SEC-MALLS analysis. However unlike the monomeric EK/RD variant, the elution profile of this peak was strongly asymmetric and showed concentration dependence in both elution position and the calculated M_*w*_. MALLS analysis showed the M_w_ varied across the peak reaching a maximum of 30,612 Da. The M_*w*_ then decreases to that of a monomer as the peak tails towards the elution position of the monomeric EK/RD construct (Fig. 3a). This behaviour is characteristic of a protein undergoing a concentration dependent self-association on the time-scale of the separation. As the protein dilutes, the monomeric species is retarded in the column resulting in a trailing edge to the peak. The reported M_*w*_ is an average of species present in each fraction and as such does not correspond with a discrete species. The maximum calculated M_*w*_ at the peak demonstrates the presence of a species much larger than would be expected for a dimer.

To further characterise the self-association of the Bbox interface we undertook SV-AUC and sedimentation equilibrium analytical ultracentrifugation (SE-AUC) experiments. In SV-AUC RhT5 88–261dCC showed a clear concentration dependence in the profiles of c(S) analysis. At 5 μM the protein migrates as a single broad peak at 1.7 S (Fig. 3b and Table S5). As the concentration is increased this resolves into two peaks at approximately 1.7 S and 2.6 S. The half-point, where the slower and faster peaks have approximately equal areas, occurs at a concentration between 20 and 40 μM.

Multi-speed SE-AUC analysis was used to confirm the presence of a monomer-trimer equilibrium and to determine the affinity of this interaction (Fig. 3c and Fig. S6). Data were first fit to a species model where the mass of a monomer was fixed (12,354 Da) and the mass of the multimer was allowed to float. This resulted in a mass for the multimer of 35,050 Da that compares well to the trimer mass of 37,062 Da. Data were then progressively fit to monomer-dimer, monomer-trimer and monomer-tetramer models in SEDPHAT. The best fit to the data was provided by a monomer-trimer model (Chi-square 0.22) with a dissociation constant of 0.98 nM^2^ corresponding to a half-point of the transition between monomer and trimer of 32 μM in agreement with that seen in the SV analysis.

Small angle X-ray scattering of the trimeric RhT5 88–216dCC construct was undertaken to determine the low-resolution structure. Data were collected between 60–480 μM (Fig. 3d). Across all protein concentrations of RhT5 88–261dCC the Rg, D_max_, and molar mass (MM) predicted from I(0) increases as a function of protein concentration (Table S7). At the two highest measured protein concentrations of 240 and 480 μM these variables plateau. Based on the sedimentation data, at these concentrations the protein is largely in the trimeric form (83% and 89% respectively) that will dominate the scattering curve. In addition, examination of the I(0) and Rg are consistent with the trimeric species.

*Ab initio* bead models generated with DAMMIF without symmetry constraints show a propensity to form a triangular assembly. Therefore, subsequent models were generated with P3 symmetry. The resulting models all showed a trimeric spoke pattern with an overall Χ^2^ of 1.9 (Fig. 3e). This arrangement is consistent with the Bbox domains assembling at the centre and the three helix bundles extending away from the vertex.

### The RING domain contributes to assembly

**Figure 4:**
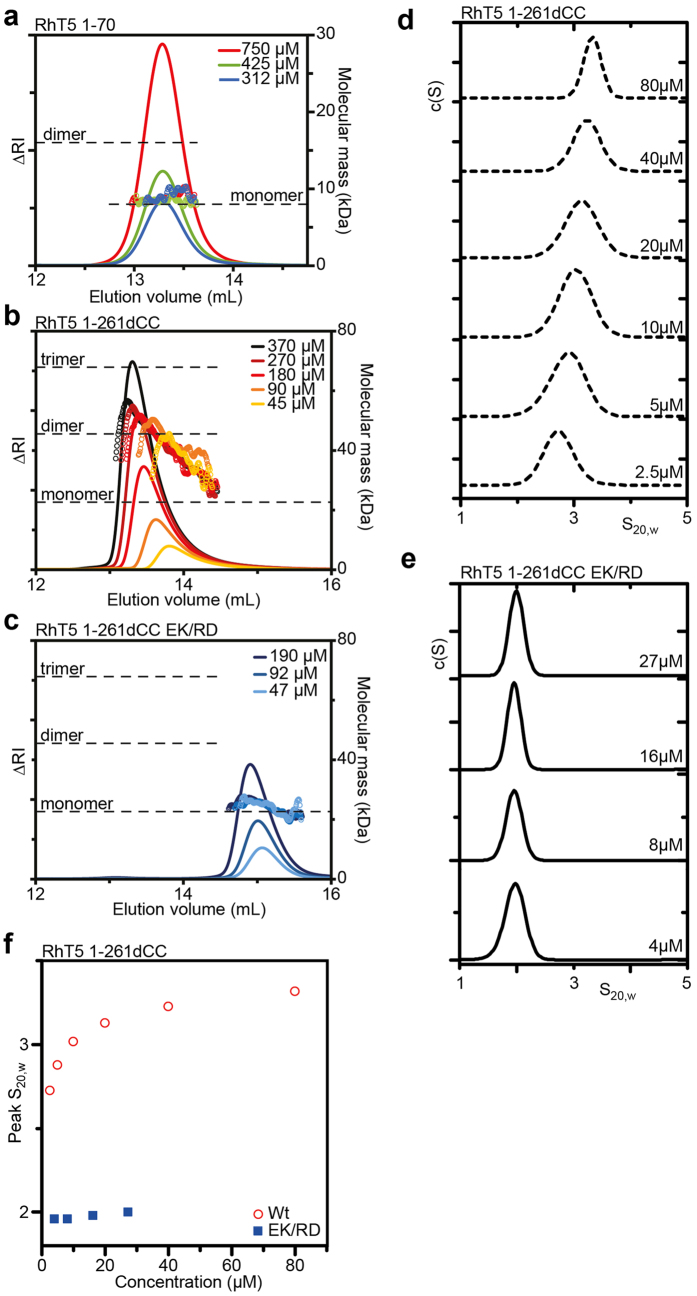
The Trim5α Bbox supports assembly of the RING domain. SEC-MALLS analysis of (**a**) Trim5α RING domain (RhT5 1–70) (**b**) RhT5 1–261dCC Wt and (**c**) RhT5 1–261dCC EK/RD. SV-AUC c(S) analysis of (**d**) RhT5 1–261dCC and (**e)** RhT5 1–261dCC EK/RD. (**f**) Sedimentation isotherm of RhT5 1–261dCC EK/RD and Wt.

To investigate the effect of the RING domain on assembly we assessed the self-associative properties of RhT5 1–261dCC that includes the RING domain. As has been shown previously the RING domain of Trim5α is monomeric in solution^22^. A SEC-MALLS analysis of RhT5 1–70 shows a single peak eluting at 13.2 mL on a Superdex 75 10/300 column. This peak has an invariant M_*w*_ of 8.3 kDa and no concentration dependent oligomerisation (Fig. 4a). By comparison, SEC-MALLS analysis of RhT5 1–261dCC showed a strong concentration dependence in elution position and a clear asymmetric peak shape, as was seen for RhT5 88–261dCC (Fig. 4b). The molecular weight at the peak of the highest concentration sample was ~58 kDa, approaching that of a trimer (67.8 kDa) and greatly exceeding that predicted for a dimeric species. The late eluting tail of the peak had a M_w_ nearing that of the monomer consistent with the protein existing in a reversible equilibrium.

Addition of the E120K/R121D mutation to the RhT5 1–261dCC abrogates much of the self-association seen in the Wt construct (Fig. 4c). However, the protein elutes earlier with increasing protein concentration indicating a propensity for weak self-association still remains. This association was not seen for the RhT5 88–261dCC EK/RD construct.

Sedimentation velocity-AUC experiments of RhT5 1–261dCC show a concentration dependent shift in S-value with a maximum of 3.3 S (Fig. 4d and Table S8). This is compared with an S-value of 2 S for RhT5 1–261dCC EK/RD that showed no concentration dependence over the concentration range measured (4–27 μM) (Fig. 4e). At the lowest concentration of RhT5 1–261dCC (2.5 μM), an S of 2.7 S suggests a substantial proportion of the protein is in the trimeric form demonstrating that the interaction is considerably tighter than the Bbox alone. Analysis of the weight-averaged S isotherm (Fig. 4f), fit to a monomer-trimer equilibrium, estimates the half point of the interaction at ~2 μM corresponding to a Kd of 3 pM^2^. This is approximately 10 times stronger than the Bbox interaction alone and implicates elements from the RING domain and L1 region in assembly.

### Assembly supports increased ubiquitylation activity

The RING domain of Trim5α, while not essential to block infection, greatly enhances the potency of restriction^11^,^23^. This potency is evident in the ability to block reverse transcription. Deletion or disruption of the Trim5α RING domain or inhibition of ubiquitylation delays the block to infection until after RT has been completed^4^,^7^,^24^.

**Figure 5:**
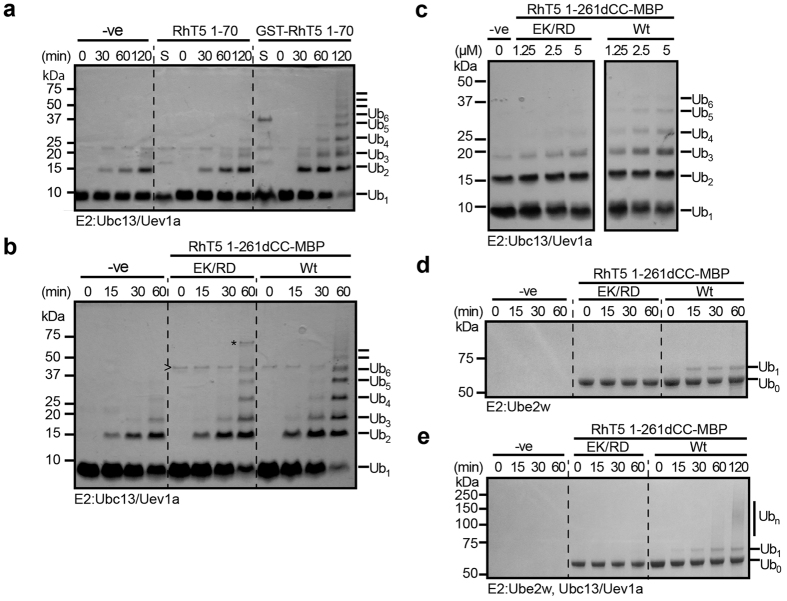
Assembly supports ubiquitylation. Ubiquitylation assays showing ubiquitin chains formed by the E2 enzymes Ubc13/Uev1a and Ube2W in the presence of various Trim5α constructs containing the RING domain. (**a**) Dimerisation of the Trim5α RING domain by fusion to GST promotes ubiquitylation. The negative control lacks E3 ubiquitin ligase to account for background activity of Ub13/Uev1a. Lane S shows unprecipitated reaction components prior to initiation of the reaction. (**b**) Ubiquitylation assays containing either RhT5 1–261dCC-MBP EK/RD or Wt were sampled and analysed by SDS-PAGE. The marked bands correspond to MBP(>) and RhT5 1–261dCC-MBP(*) that failed to precipitate. (**c**) Ubiquitylation assays with varying concentrations of either RhT5 1–261dCC-MBP EK/RD or Wt between 1.25–5 μM were sampled at 30 min and analysed by SDS-PAGE. (**d**) Ubiquitylation assay with the E2 enzyme Ube2W. (**e**) Ubiquitylation assay with both Ube2W and Ubc13/Uev1a. In (**d**,**e**) indicated bands represent Ub fused to RhT5 1–261dCC-MBP.

To assess the effect of self-association on Trim5α activity, ubiquitylation assays were carried out using the heterodimeric E2 enzyme Ubc13/Uev1a that combines with Trim5α to activate innate immune signalling^9^. This E2 enzyme catalyzes the formation of unanchored K63-linked ubiquitin chains that activate the TAK1 kinase complex^9^. Assay reactions were initiated by the addition of ATP and consisted of Ube1, Ubc13/Uev1a, and selected Trim5α constructs containing the RING domain. For each assay a control reaction without Trim5α was used to assess the background activity of the Ubc13/Uev1a E2 complex^25^ (Fig. 5a lanes 1–4). Assays with the Trim5α RING domain alone (RhT5 1–70) did not result in chain formation above the background levels observed the absence of this construct (Fig. 5a). Fusion of the Trim5α RING domain to GST to promote formation of a dimer, resulted in a robust increase in activity consistent with a requirement for dimerization as previously described^22^ (Fig. 5a lanes 9–12).

To establish whether the assembly of the Bbox domain could support the activation of ubiquitylation by the Trim5α RING domain we assayed the ubiquitylation activity of RhT5 1–261dCC. To aid in protein expression and purification we constructed a version with MBP fused to the C-terminus that was used for ubiquitylation assays. Ubiquitylation assays with RhT5 1–261dCC-MBP demonstrated a dramatic increase in the ubiquitylation activity in the presence of the Bbox compared to the background (Fig. 5b compare lanes 1–4 (−ve) to lanes 9–12 (Wt)). This restored activity to levels comparable to that seen with the GST-RING fusion with chains longer that Ub_5_ visible after 30 min and longer than Ub_8_ after 60 min. Unexpectedly, RhT5 1–261dCC-MBP carrying the EK/RD Bbox mutation that blocks assembly retained some activity over the negative control (Fig. 5b compare lanes 1–4 (−ve) with 5–8 (EK/RD)). However, this was greatly reduced compared to the Wt with Ub_3_ chains present after 30 min and a maximum length of Ub_6_ after 60 min and a reduced intensity evident for each band.

Given the weak self-association of RhT5 1–261dCC EK/RD observed on the SEC-MALLS we sought to examine if there was a difference in the concentration dependent activation of ubiquitylation between the EK/RD and Wt constructs. Ubiquitylation assays of both constructs between 1.25 μM and 5 μM sampled at 30 min show concentration dependence in activity (Fig. 5c). At 1.25 μM the RhT5 1–261dCC EK/RD mutant shows little activity above the control with a slight increase in the Ub3 species. By comparison the Wt protein shows a strong increase in Ub3 and the appearance of Ub4 and Ub5 species. As the concentration is increased both constructs show an increase in activity as expected for an increase in the amount of enzyme present. However, the activity of the Wt protein is greater than the EK/RD mutant.

A recent study has shown that the E2 enzyme Ube2W is also required to block reverse transcription^24^. Ube2W has been shown to attach mono-ubiquitin to the Trim5α RING domain which is proposed to act as an anchor for the tethering of K63 Ub chains. We repeated the assay, comparing the activity of the Wt and the EK/RD mutant of RhT5 1–261dCC-MBP in the presence of Ube2W. The assay was carried out as described for Ubc13/Uev1a except that samples were not precipitated prior to SDS-PAGE. Within 15 min a band corresponding to mono-ubiquitylated RhT5 1–261dCC is visible in the Wt samples that increases after 30 and 60 min. In contrast only a faint band is present in the EK/RD samples (Fig. 5d and Fig. S9) showing a clear difference in the Ube2W mediated ubiquitylation activity of Wt and EK/RD mutant as well. Inclusion of both Ube2W and Ubc13/Uev1a resulted in a high molecular weight species, visible after 60 mins, that is not present in the EK/RD mutant (Fig. 5e, compare lanes 8 and 12). A Wt sample at 120 mins shows the formation of larger chains (Fig. 5e, lane 13), consistent with the additional chain formation seen by Fletcher *et al.*^24^. Together with the previous ubiquitin assays these experiments demonstrate an assembly mediated increase in ubiquitylation activity by the Trim5α RING domain.

## Discussion

Recognition of the intact capsid lattice is central to restriction of retroviral infection by Trim5α. While the C-terminal PRY/SPRY domain is responsible for direct recognition of the viral capsid protein, the functionality of the RBCC components are required for efficient restriction. Chimeric Trim5α constructs containing the Trim21 RING domain have been shown to assemble into a large hexagonal array that is complementary to the hexameric capsid lattice providing a mechanism for recognition of the capsid array^16^. To investigate the assembly of Trim5α we have examined the RING and Bbox domains of Trim5α and the ability of these domains to promote assembly in the context of a truncated ‘monomeric’ form of the Trim5α protein dimer.

Analysis the RhT5 88–261dCC EK/RD construct shows a compact folded domain with properties that are equivalent to those predicted from our structural model. This is consistent with our hypothesis that a hydrophobic core extends from the central sheet of the Bbox domain into the coiled-coil hairpin. As such, this construct avoids problems associated with having binding interfaces at each end of the coiled-coil that form polymers. The wild type construct showed a clear concentration dependent assembly that could be attributed to a monomer-trimer equilibrium. Models generated from SAXS analysis show a trimeric spoke arrangement, consistent with the Bbox being at the centre of the three-fold axis and the coiled-coil extending away (Fig. 2e). With equivalent interactions at either end of the antiparallel coiled-coil this trimeric interface would facilitate the assembly of the Trim5α hexagonal array with the Bbox domain located at the three-fold vertices as proposed^17^.

Addition of the RING domain strengthens the interaction increasing the dissociation constant from 0.98 nM^2^ to 3 pM^2^. One possible reason for this increase is the four-helix bundle that forms upon RING domain dimerisation^22^ providing an additional assembly interface. The increase in affinity is also consistent with previous reports that the RING domain strengthens the association of Trim proteins^26^ and enhances Trim5α binding to HIV-1. However, the affinity of RING dimersation alone is not sufficient for oligomerisation, only in the presence of the Bbox domain can it support assembly. In addition, the E120K/R121D mutation produces Trim5α which is unable to block infection, further indicating that *in vivo* the RING domain is insufficient for assembly^18^.

While the RING domain of Trim5α is not required to block infection it does mediate the early block to restriction and is required to block reverse transcription^4^,^24^. This activity is attributed to its ubiquitylation activity as proteasome inhibitors circumvent this block to infection. Similarly depletion of the E2 ubiquitin conjugating enzymes Ube2W or Ubc13/Uev1a that prevent ubiquitin chain formation^9^,^24^ or the incorporation of deubiquitylases (DUBs) at the N-terminus of Trim5α restore reverse transcription^27^.

In the context of full length Trim5α, the RING domains are held at either end of the Bbox-coiled coil dimer. The RING domain is only active upon dimerization^22^, meaning that a single Trim5α dimer is inactive as an E3 ubiquitin ligase. While ubiquitylation of Trim5α in cells is not dependent upon the presence of virus, interaction with the capsid lattice has been shown to increase the formation of ubiquitin chains^9^. The templated assembly of Trim5α on the capsid lattice^16^, supported by the trimeric Bbox interaction presented here, provides a possible mechanism to increase ubiquitylation activity in response to incoming retroviral capsid.

In support of this hypothesis, we have shown that assembly via the Bbox domain is able to support a robust increase in ubiquitylation activity, both in the formation of unanchored chains with Ubc13/Uev1a, and anchored ubiquitin with Ube2W. Furthermore, inclusion of both E2 enzymes results in an increase in high-molecular weight ubiquitin chains as seen by Fletcher *et al.*^24^. This activity is greatly attenuated upon inclusion of the assembly blocking mutant E120K/R121D. While this construct retains a reduced level of activity, most evident in the presence of only Ubc13/Uev1a, we attribute this to a weak self-association in the presence of the RING domain. Moreover, inclusion of both Ube2W and Ubc13/Uev1a results in high-molecular weight ubiquitin chains that are not present in the non-assembling EK/RD mutant.

The activation of ubiquitylation at the three-fold interface of the Trim5α assembly requires dimerisation of the RING domain^22^. This immediately poses the question of how the third RING domain is accommodated. A mechanism where the third RING domain acts as an acceptor for ubiquitylation has been proposed by Yudina *et al.*^22^. An alternative hypothesis is that the high local concentration of RING domains at the three-fold vertices acts to amplify activity and that the third RING domain is a bystander that is ready to participate in subsequent reactions.

In this study we have shown that the RING, Bbox, and coiled-coil domains of Trim5α contribute to the trimeric interface that drives assembly of the hexagonal lattice. There are currently in excess of 100 members of the TRIM protein family that are involved in diverse cellular processes^28^. These proteins all share the RING-Bbox-coiled-coil architecture. The coiled-coil domain has been shown to be an antiparallel dimer in all studied Trim proteins^17^,^29^,^30^, suggesting that a similar arrangement of the RING and Bbox domains is present. Consequently, it is likely that higher order assembly is required for activation of ubiquitylation in all TRIM proteins and that a similar requirement for elements from each domain drive assembly. Wide reaching biophysical studies of many TRIM proteins are required to fully understand the role self-association plays in the many processes where they have a critical function.

## Methods

### Cloning

DNA encoding RhT5 1–261Δ155–228 (RhT5 1–261dCC) was codon optimised and synthesised. The coding sequences for RhT5 1–70, RhT5 94–141, RhT5 1–261dCC, and RhT5 88–261dCC were amplified by PCR and cloned into pET47b (N-terminal His-tag) or pET49b (N-terminal GST-tag) by a ligation independent cloning method^31^. The resulting constructs contain an N-terminal purification tag with 3 C-protease cleavage site. Two versions of RhT5 1–261dCC were generated in pET47b based vectors. RhT5 1–261dCC contains a 3 C protease cleavable C-terminal MBP tag. RhT5 1–261dCC-MBP contains a 3 C protease cleavable N-terminal His tag and non-cleavable C-terminal MBP tag. The E120K/R121D variants were generated by whole plasmid PCR using the wild type plasmid as templates.

### Protein expression and purification

Briefly, RhT5 1–70, RhT5 94–141, RhT5 88–261dCC, and RhT5 88–261dCC E120K/R121D were expressed in *E. coli* BL21(DE3) and the proteins purified by IMAC. Cells were grown in LB, supplemented with 25 mM Na_2_HPO_4_, 25 mM KH_2_PO_4_, 50 mM NH_4_Cl, 5 mM NaSO_4_, 0.5% v/v glycerol, 0.05% w/v glucose, 2 mM MgSO_4_ and 100 μM ZnCl_2_. Cells were grown at 37 °C to approximately 1.2 OD prior to being transferred to 18 °C and protein expression induced with 1 mM Isopropyl β-D-1-thiogalactopyranoside (IPTG) for 16 hours. Cells were lysed in 20 mM Tris-HCl pH 7.8, 300 mM NaCl, 0.5 mM TCEP, 20 mM imidazole and the protein purified by immobilised metal affinity chromatography. The N-terminal purification tags were removed by cleavage with 3 C-protease prior to further purification by anion exchange, and size-exclusion chromatography (Superdex 75 16/60).

All RhT5 1–261dCC constructs (Wt and EK/RD) were expressed in LB broth supplemented with 100 μM ZnCl_2_. Cells were grown at 37 °C to an optical density of 0.6–0.8, then transferred to 18 °C. Expression was induced with the addition of 1 mM IPTG for 16 hours. Cells were lysed in 20 mM Tris-HCl pH 7.8, 200 mM NaCl, 0.5 mM TCEP and 10% glycerol. RhT5 1–261dCC-MBP with an N-terminal His-tag were purified by amylose resin affinity chromatography, then immobilised metal affinity chromatography, followed by SEC on a Superdex 200 16/60 column. RhT5 1–261dCC with cleavable C-terminal MBP was purified by amylose resin affinity chromatography with on column 3 C protease cleavage overnight, followed by anion exchange chromatography.

### SEC-MALLS

Size-exclusion chromatography coupled to multi-angle laser light scattering (SEC-MALLS) was used to determine the solution molecular weight and assess protein heterogeneity. Samples (100 μL) were applied to either a Superdex 75 10/300 GL (RhT5 1–70, RhT5 88–261dCC) or Superdex S200 Increase 10/300 GL (RhT5 1–261dCC) column equilibrated in 10 mM Tris/HCl pH 7.8, 150 mM NaCl, 0.1 mM TCEP mounted on a Dionex HPLC with a PSS SLD7000 7-angle MALLS detector and Shodex RI-101 differential refractive index detector. The weight average molecular weight was determined using PSS winGPC Unichrom software.

### SAXS

Samples for small angle X-ray scattering were dialysed extensively against 10 mM Tris-HCl pH 7.8, 150 mM NaCl, 0.1 mM TCEP. Data for all constructs were collected at the Australian Synchrotron SAXS/WAXS beamline at a wavelength of 1.13 Å with a camera length of 1.6 m covering a momentum transfer range of 0.007 < q < 0.6 Å^−1^ (q = 4πsin(θ)/λ). Data were collected on a serial dilution of each construct in capillary and images were processed using Scatterbrain and PRIMUS.

SAXS data were further analysed using programs in the ASTAS package^32^. *Ab initio* models were produced in GASBOR and DAMMIF and consensus models generated with DAMAVER.

### Ubiquitin assay

Each assay reaction contained 50 mM Tris/HCl, pH 7.8, 150 mM NaCl, 10 mM MgCl_2_, 0.1 mM TCEP, 120 nM Ube1, 5 μM E2, 125 μM ubiquitin, and equimolar concentrations of TRIM5α constructs. All Ub assay components except ATP were mixed on ice. ATP was added to initiate the reaction and the reaction mixture was incubated at 37 °C for the duration of the assay. For Ubc13/Uev1a assays, reactions were quenched with a 1:1 ratio of 10 mM sodium acetate pH 4.5 and incubated at 60 °C for 10 min to precipitate all proteins other than free ubiquitin chains. For Ube2W containing assays, reactions were quenched with a 1:1 ratio of SDS loading dye. Quenched reactions were then assessed by SDS-PAGE.

### Analytical ultracentrifugation

All analytical ultracentrifugation experiments were carried out at 20 °C (293 K) using a Beckman Coulter Model XL-I instrument equipped with an absorbance optical system. Samples were loaded into double-sector quartz cells and mounted into a Beckman Coulter eight-hole An-50 Ti rotor. Prior to sedimentation samples were matched to a buffer containing 10 mM Tris/HCl pH 7.8, 150 mM NaCl either by extensive dialysis or SEC. SEDNTERP^33^ was used to calculate a solvent density of 1.00499 gmL^−1^, viscosity of 0.01021 cp. Protein partial specific volumes were calculated from the protein sequence, RhT5 88–261dCC and RhT5 88–261dCC E120K/R121D were 0.732 mLg^−1^. The Bbox only construct comprising residues 94–141, 0.719 mLg^−1^ and the RhT5 1–261dCC constructs 0.737 mLg^−1^ for Wt and E120K/R121D.

Sedimentation velocity of the RhT5 94–141 and RhT5 88–261dCC constructs were measured at 50 krpm and RhT5 1–261dCC constructs at 38 krpm. Data were analysed using the c(S) or c(M) distributions in SEDFIT^34^. To estimate the half point transition for the monomer-trimer interaction isotherm analysis of the integrated c(s) was carried out using the monomer-trimer model in SEDPHAT^35^. Sedimentation equilibrium experiments were carried out at three concentrations (0.125, 0.25, 0.5 mg/ml) of RhT5 88–261dCC using 110 μL of sample and 120 μL reference and centrifuged at 14,000, 18,000, and 22,000 rpm. Data were collected at a wavelength of 280 nm, with a step size of 0.001 cm, and averaging of 20 measurements at each step. The data were analysed using SEDPHAT and a monomer-trimer self-association model was chosen.

## Additional Information

**How to cite this article**: Keown, J. R. *et al.* Characterisation of assembly and ubiquitylation by the RBCC motif of Trim5α. *Sci. Rep.* **6**, 26837; doi: 10.1038/srep26837 (2016).

## Supplementary Material

Supplementary Information
