# Supplementary material for: Characterisation of assembly and ubiquitylation by the RBCC motif of Trim5α
Source: Sci Rep. 2016 May 27;6:26837. doi: 10.1038/srep26837 (PMC4882581; doi:10.1038/srep26837)
Supplement: Supplementary Information [file srep26837-s1.pdf]

SUPPLEMENTARY

Characterisation of assembly and ubiquitylation by the RBCC motif of Trim5α

Jeremy R. Keown[1], Joy X. Yang[1], Jordan Douglas[1], David C. Goldstone[1,2,#]

[1]*School of Biological Sciences, University of Auckland, Auckland, New Zealand*

[2]*Associate Investigator Maurice Wilkins Centre for Molecular Biodiscovery*

[#]To whom correspondence should be addressed

Telephone: +64 9 923 4607

E-mail: d.goldstone@auckland.ac.nz

Protein concentration (μM)	Integrated c(s) ($S_{20,w}$)	f/f_0	Weight averaged MM (kDa)	Peak 1 Abundance (%)	Peak 2 Abundance (%)
200	1.54	1.24	12.1	58.3	41.7
100	1.35	1.22	9.6	70.4	29.6
50	1.23	1.34	9.7	84.0	16.0
25	1.17	1.2	7.6	100	0
12.5	1.17	1.29	8.4	100	0

Table S1. Sedimentation velocity parameters for RhT5 94-141.

Protein concentration (μM)	Integrated c(s) ($S_{20,w}$)	f/f_0	Weight averaged MM (kDa)	Peak 1 Abundance (%)	Peak 2 Abundance (%)
80	1.56	1.259	12.9	100	0
40	1.55	1.295	13.3	100	0
20	1.54	1.307	13.4	100	0

Table S2. Calculated parameters for AUC data on RhT5 88-261dCC E120K/R121D The data shows a single species with a mass of ~13 kDa, corresponding to the expected molecular weight for a monomer.

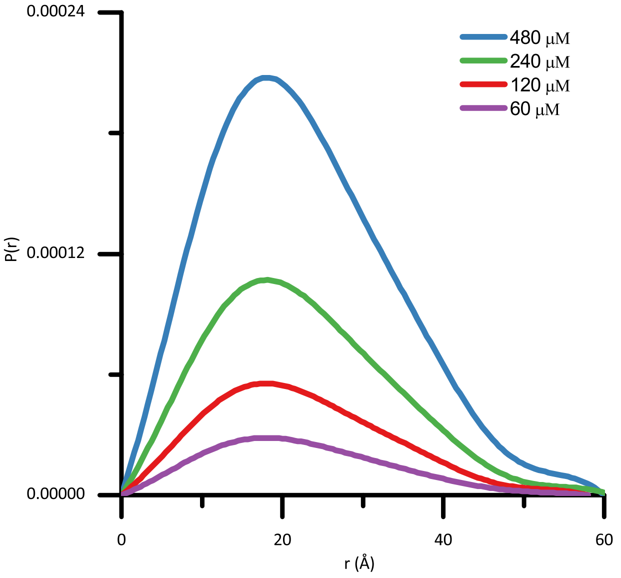

Figure S3. Pair-distribution plot for RhT5 88-261dCC E120K/R121D. The p(r) distribution shows a negatively skewed distribution with higher proportion of shorter distances, suggesting a slightly elongated globular protein.

Protein concentration (µM)	Radius of gyration (Å)	Porod volume ($Å^3$)	I(0)	D_{max} (Å)	MM predicted from I(0) (kDa)
480	17.3	14,988	0.072	60	15.41
240	17.3	14,972	0.037	61	15.83
120	17.3	14,736	0.019	58	16.26
60	17.5	14,822	0.010	58	17.12
Predicted	16.6	-	-	59.1	12.4

Table S4 SAXS parameters for RhT5 88-261dCC. The SAXS data shows no concentration dependence in Rg, Porod volume, or D_{max}. The predicted Rg, D_{max}, and MM were calculated with HYDROPRO for an atomic model comprising resides P88-K154, Y229-N261 from structure of RhTrim5α BBox coiled-coil (PDBid: 4TN3). This model lacks the four residue linker between K154-Y229.

Protein concentration (µM)	Integrated c(s) ($S_{20,w}$)	f/f_0	Weight averaged MM (kDa)	Peak 1 Abundance (%)	Peak 2 Abundance (%)
160	2.40	1.30	28.1	14.4	85.6
120	2.37	1.32	-	18.0	82.0
80	2.30	1.28	-	28.8	71.2
40	2.17	1.28	-	38.0	62.0
20	2.03	1.19	-	52.5	47.5
10	1.90	1.25	-	100	0
5	1.76	1.22	15.4	100	0

Table S5. AUC parameters for RhT5 88-261dCC. Strong concentration dependence is seen in the integrated $S_{20,w}$ covering both sedimentation peaks.

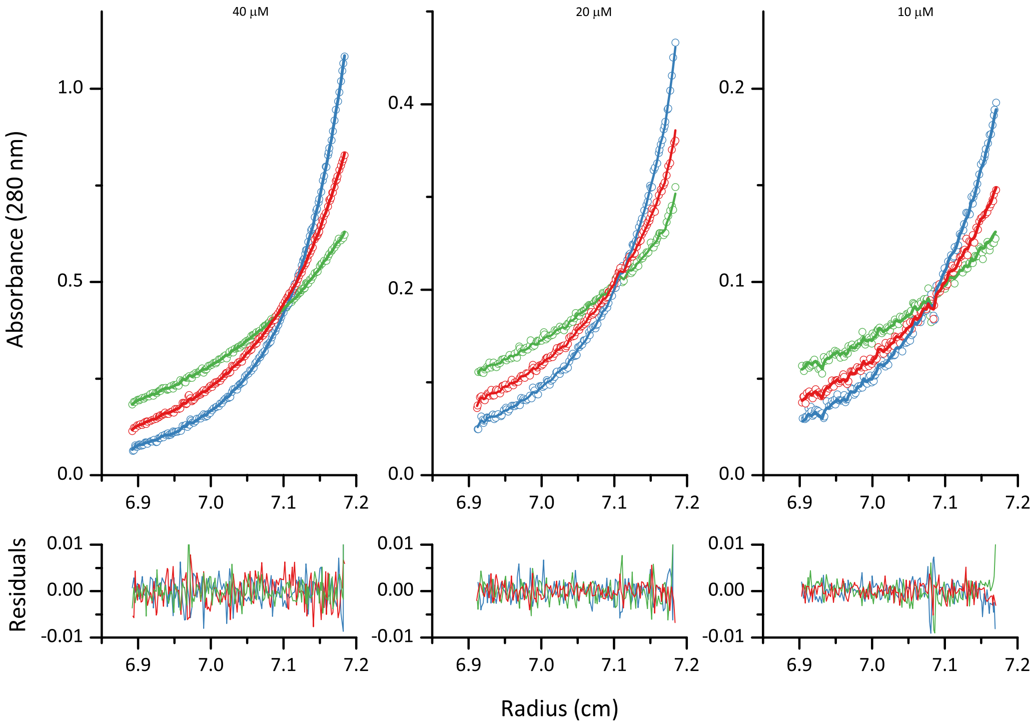

Figure S6. Sedimentation equilibrium of RhT5 88-261dCC at 10 µM, 20 µM, and 40 µM. The data were fit to a monomer-trimer self association model as implimented in SEDPHAT. Samples were centrifuged at 14,000 rpm (green), 18,000 rpm (red) and 22,000 rpm (blue). Absorbance was measured at 280 nm. Time and radial independent noise reduction was applied.

Protein concentration (μM)	Radius of gyration (Å)	Porod volume ($Å^3$)	I(0)	D_{max} (Å)	MM predicted from I(0) (kDa)
480	25.5	51,450	0.169	89.4	36.16
240	25.4	47,281	0.083	89.1	35.52
120	25.4	42,596	0.041	88.7	35.09
60	24.6	36,184	0.019	86.1	32.53

Table S7. A table of SAXS data parameters is presented here (Bottom). The Rg and D_{max} plateau at the highest two concentrations.

Protein construct	Protein concentration (µM)	Integrated c(s) ($S_{20,w}$)	f/f_0	Weight averaged MM (kDa)
RhT5 1-261 dCC E120K/R121D	27	2.00	1.41	23.0
	16	1.98	1.42	22.7
	8	1.96	1.48	23.8
	4	1.96	1.46	23.5

Protein construct	Protein concentration (µM)	Integrated c(s) ($S_{20,w}$)	f/f_0	Weight averaged MM (kDa)
RhT5 1-261 dCC	80	3.32	1.36	48.8
	40	3.23	1.41	46.8
	20	3.13	1.43	45.5
	10	3.02	1.40	41.8
	5	2.88	1.4	38.8
	2.5	2.73	1.49	39.5

Table S8. AUC Data parameters for RhT5 1-261dCC and RhT5 1-261dCC EK/RD.

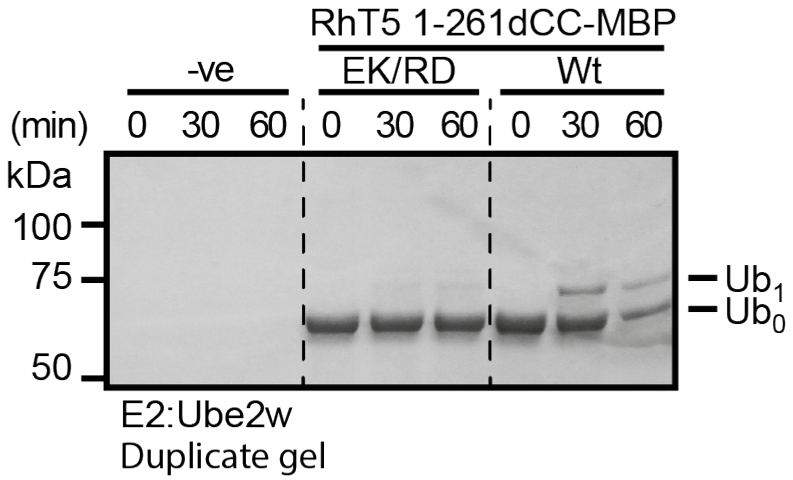

Figure S9. Ubiquitylation assay in the presence of Ube2w. Duplicate gel of Figure 5d highlighting faint band present in the EK/RD sample. Ub0 and Ub1 bands correspond to unconjugated and ubiquitin conjugated bands of RhT5 1-261dCC-MBP respectively.
